# Stroke Risk Factors of Stroke Patients in China: A Nationwide Community-Based Cross-Sectional Study

**DOI:** 10.3390/ijerph19084807

**Published:** 2022-04-15

**Authors:** Jingyi Chen, Qianrang Zhu, Lianlong Yu, Yuqian Li, Shanshan Jia, Jian Zhang

**Affiliations:** 1 National Institute for Nutrition and Health, Chinese Center for Disease Control and Prevention, Beijing 100050, China; jingyich@126.com (J.C.); qianrang.zhu@wur.nl (Q.Z.); lianlong00a@163.com (L.Y.); cnu_lyq@126.com (Y.L.); jssky.good@163.com (S.J.); 2 Shandong Center for Disease Control and Prevention, Ji’nan 250000, China; 3 Key Laboratory of Trace Element Nutrition of National Health Commission (NHC), Beijing 100050, China

**Keywords:** stroke risk factors, stroke patients, China

## Abstract

Background: Stroke is the leading cause of death in China, and its burdens are rapidly increasing. The prevalence and control of stroke risk factors among stroke patients in China are unknown. Objective: We investigated the stroke risk factors of stroke patients in China. Design: We examined stroke risk factors in 6580 stroke patients aged 18 years or older in the China National Chronic Diseases and Nutrition Surveillance of Adults (2015–2017). With regard to the basic characteristics of the study participants, categorical variables were described as frequency (percent). The chi-square test was used to analyze the difference between men and women. The multivariate logistic regression model was used in the multivariate analysis. Results: Among the 6580 stroke patients, hypertension was the most common stroke risk factor identified in most cases (78.51%), followed by overweight or obesity (61.58%), dyslipidemia (54.38%), smoking (24.04%), diabetes (21.75%), family history of stroke (17.43%), lack of exercise (16.35%), and atrial fibrillation (4.47%). Drinking stroke patients had a lower rate of hypertension, diabetes, and dyslipidemia. Patients with hyperuricemia had a higher rate of hypertension and dyslipidemia than no-hyperuricemia patients. The hypertension awareness, treatment, and control rates among hypertension stroke patients were 73.62%, 70.19%, and 17.79%, respectively. The diabetes awareness, treatment, and control rates among diabetes patients were 69.74%, 65.83%, and 34.59%, respectively. The dyslipidemia awareness, treatment, and control rates among dyslipidemia patients were 42.37%, 29.4%, and 20.07%, respectively. Among treated hypertension patients, the rates of taking medicine as medically advised, controlled diet, increased exercise, and blood pressure monitoring were 91.31%, 58.88%, 45.78%, and 73.99%, respectively. Among treated diabetes patients, the rates of oral antidiabetic medications, insulin injection, diet control, and blood glucose monitoring were 78.24%, 34.71%, 85.77%, and 78.24%, respectively. Among treated dyslipidemic patients, the rate of taking medicine as medical advice, controlled diet, increased exercise, and regular blood lipid monitoring was 80.61%, 77.57%, 56.46%, and 40.3%, respectively. Conclusions: The most common risk factors for community stroke patients in China are hypertension, dyslipidemia, and overweight or obesity. The stroke community patients’ suboptimal awareness and treatment of hypertension, and suboptimal awareness, treatment, and control of diabetes, and dyslipidemia are significant problems in China.

## 1. Introduction

Stroke is the second leading cause of death worldwide [1]. There are approximately 2.5 million new stroke cases in China per year [2]. Stroke patients are at high risk for recurrent stroke or other major vascular events [3,4,5,6,7]. Multiple studies have shown that control of modifiable risk factors (hypertension, diabetes, smoking, dyslipidemia, obesity and others) can reduce the risk of stroke recurrence [8,9,10]. Guidelines for the prevention of stroke in patients with stroke and transient ischemic attack also emphasize the importance of risk factor control [11]. However, previous studies have found that the control of risk factors for stroke patients in China is not ideal [12,13]. Hypertension, diabetes, and dyslipidemia are major risk factors for cardiovascular diseases including stroke [14,15,16].

Hypertension is the crucial modifiable risk factor for stroke, and controlling blood pressure can effectively reduce recurrence of stroke [6,17]. The hypertension control rate after stroke has been less well studied. In a survey of patients in the China National Stroke Registry II, the prevalence of hypertension was 64.9% at baseline, and 69.84% after taking antihypertensive medications for 3 months after discharge [12].

Diabetes is a modifiable risk factor for ischemic stroke. The baseline prevalence of diabetes of patients in the China National Stroke Registry II was 20.56%, and 71.82% of patients took diabetes medications at 3 months [12]. In a study about the stroke health manager, 62.1% of the control group were ischemic stroke patients and 66.0% were the intervention group and after three months of intervention, the blood glucose was from 8.00 mmol/L to 7.3 mmol/L in the intervention group and from 8.11 mmol/L to 8.05 mmol/L in the control group [18].

Dyslipidemia is the main pathophysiological cause of atherosclerosis leading to stenosis and blockage of arteries and vessels and easily leading to stroke. Studies have shown that lipid-lowering statins can reduce the risk of myocardial infarction and stroke [19,20]. A total of 37.85% of patients had persistently taken statins in the China National Stroke Registry II at 3 months after discharge [12]. In a study about the stroke health manager, 88.7% of stroke patients in the control group and 95.2% in the invitation group had persistently taken statins, and the control group LDL-C was from 2.50 mmol/L to 2.62 mmol/L; intervention group LDL-C was from 2.62 mmol/L to 2.07 mmol/L.

There is some research about stroke risk factors in the general population and other studies focus on patients in hospitals in the acute clinical phase [21]. However, the longer term issues need to be better researched, and there are still no nationwide reports on the prevalence and control of stroke risk factors in the community of stroke patients over age 18. This study focused on the epidemiology of stroke risk, awareness rate, treatment rate, control rate of hypertension, diabetes, and dyslipidemia in Chinese community stroke patients, to provide policy recommendations for stroke prevention and intervention in China.

## 2. Materials and Methods

### 2.1. Study Design and Participants

The data used in this study were obtained from China National Chronic Diseases and Nutrition Surveillance of Adults (2015–2017) carried out in 31 provinces in China. The China National Chronic Diseases and Nutrition Surveillance of Adults (2015–2017) is a nationally representative cross-sectional study conducted by the Chinese Center for Disease Control and Prevention to assess food and nutrient intakes, current chronic diseases status, and health behaviors of the Chinese population. A stratified, multistage probability cluster sampling design was used to select subjects. Further information is described in other articles [22].

This study included stroke patients aged 18 years or older who had complete data on medical history, physical examination, and laboratory results in China National Chronic Diseases and Nutrition Surveillance of Adults (2015–2017). We further excluded participants without self-reported stroke-related information from the data collected by questionnaires. Finally, a total of 6580 participants were enrolled in this study.

All procedures involving human subjects were approved by the Ethics Committee of the Chinese Center for Disease Control and Prevention (grant no. 201519-B). Written informed consent was obtained from all participants.

### 2.2. Data Collection and Definition

Research objects were selected by multi-stage cluster random sampling. The sampling was divided into four stages. In the first and second stages, the sampling method was systematic sampling with population size ranking. The sampling method of stage 3 and stage 4 was simple random sampling. In the first stage, 3 townships, streets, and regiments were selected from each monitoring site. In the second stage, 2 administrative villages or neighborhood committees and associations were selected from each township or street or regiment in the first stage. In the third stage, every 60 households were divided into a group that was randomly selected from each administrative village, neighborhood committee, or company selected in the second stage. The fourth stage: 45 households were selected from each group selected in the third stage for investigation; 20 households were selected as dietary survey households, and the dietary survey was based on the personal questionnaire, physical measurement, and blood sample collection. The remaining 25 households were selected as non-dietary survey households.

The face-to-face questionnaire conducted by well-trained public health physicians was designed to obtain comprehensive information about the participants. The questionnaire collected information on demographic characteristics including age, gender, ethnicity, education, marital status, and annual household income. Lifestyle information on smoking status and alcohol drinking status was also obtained. The past medical history such as stroke, atrial fibrillation, hypertension, diabetes, and dyslipidemia was obtained by self-report. Family history of diabetes, hypertension, hyperlipidemia, and stroke was also recorded.

Physical examinations were performed by trained medical staff according to standardized procedures. Bodyweight was measured using calibrated electronic digital scales with a precision of 0.01 kg, and height was measured using height measuring bars with a precision of 0.1 cm. BMI was calculated as BMI = kg/m^2^. Waist circumference was measured using non-elastic tape with a precision of 0.1 cm. After sitting quietly for 5 min, systolic and diastolic blood pressures were measured three times by an automatic measurement device (Omron HBP-1300; OMRON Healthcare, Hoofddorp, The Netherlands) with a 1 min interval between each measurement. The average of three measurements was used in the analysis. All participants were invited to provide 12 h overnight fasting blood samples. Blood samples were centrifuged and separated into plasma and serum within 0.5–1.0 h after blood collection, and frozen at −80 °C for subsequent testing. Fasting blood glucose, triglycerides, total cholesterol, high-density lipoprotein cholesterol, and low-density lipoprotein cholesterol levels were measured using a Hitachi Automatic Analyzer 7600 (Hitachi Co., Tokyo, Japan). A stroke patient was defined as if they answered “yes” to the question: “have you ever had a stroke confirmed by a professional physician”. They were further asked to recall the date or age of the first recorded diagnosis and types of stroke (ischemic stroke or hemorrhagic stroke). Participants with stroke in this cross-sectional study were non-fatal stroke survivors. BMI was grouped into four categories according to the Chinese criteria: underweight (<18.5 kg/m^2^), normal weight (≥18.5 kg/m^2^ and <24.0 kg/m^2^), overweight (≥24.0 kg/m^2^ and <28.0 kg/m^2^), or obese (≥28.0 kg/m^2^). Overweight or obese was defined as BMI ≥ 24.0 kg/m^2^.

The sample size was calculated in two layers: urban and rural (prefecture-level or above city districts, county-level cities and counties). The geographical distribution was divided into 31 layers by province. According to the above stratification factors, the total number of floors is 62 (2 × 31 = 62). The sample size was calculated by the formula *n* = $deff\frac{u^{2}p(1-p)}{d^{2}}$. The meanings and values of each parameter are as follows:

The confidence level was 95% (bilateral), corresponding *u* = 1.96;
Probability *p* was 9.7% of monitored diabetes prevalence in 2010;
*deff* value of design efficiency was set as 3;
Relative error *r* = 20%, *d* = 20% × 9.7%.

According to the above parameter values, the average sample size of each layer was about 2683 people. The average number of layers was 62, the average non-response rate was 10%, and the total sample size was about 185,000. About 612 people were investigated at each monitoring site.

A current smoker was defined as smoking every day or smoking but not every day. Alcohol use was defined as drinking within 30 days of the investigation date. A positive family history of stroke was defined as at least one first-degree family member (father, mother, or sibling) diagnosed with a stroke by a physician.

Diabetes mellitus (DM) was defined as fasting glucose level ≥ 7.0 mmol/L, any use of anti-diabetic drugs, or any self-reported history of diabetes. Hypertension was defined as an average SBP ≥ 140 mm Hg or DBP ≥ 90 mm Hg, any use of anti-hypertensive medication, or any self-reported history of hypertension.

Lack of physical activity was defined as not spending more than 30 min in medium and high-intensity physical activity in work, farming, housework, entertainment, and exercise per day.

Dyslipidemia was defined as a total cholesterol (TC) level ≥ 5.70 mmol/L, high-density lipoprotein cholesterol (HDL-C) level < 1.04 mmol/L, or current use of medication for dyslipidemia or any self-reported history of dyslipidemia

Hypertension/diabetes/dyslipidemia awareness rate was defined as the proportion of individuals who knew they had hypertension/diabetes/dyslipidemia before the survey.

Hypertension/diabetes/dyslipidemia treatment rate was defined as the proportion of people with hypertension/diabetes/dyslipidemia who have taken measures (including lifestyle changes and/or medication) to control hypertension/diabetes/dyslipidemia.

Hypertension control rate was defined as the proportion of hypertensive patients who have mean SBP < 140 mm Hg and mean DBP < 90 mm Hg.

Diabetes control rate was defined as the proportion of the diabetes population who have FPG < 7.0 mmol/L.

Dyslipidemia control rate was defined as the proportion of dyslipidemic patients who have a lipid index controlled at TC < 6.22 mmol/L and/or TG < 2.26 mmol/L and/or HDL-C > 1.04 mmol.

### 2.3. Statistical Analysis

Data analysis was carried out using SAS version 9.4 (SAS Institute Inc., Cary, NC, USA). With regard to the basic characteristics of the study participants, categorical variables were described as frequency (percent). The chi-square test was used to analyze the difference between men and women. The multivariate logistic regression model was used in multivariate analysis. Adjusted for patient characteristics (including gender, ethnicity, residents, age, marital status, and annual household income). Examine variables were: drinking, fresh vegetables, red meat, fresh fruit, and hyperuricemia.

## 3. Results

The response rate of stroke patients was 99.96%. A total of 6580 stroke patients, including 3519 males and 3061 females, were included in the analysis. The characteristics of the study participants are shown in Table 1. The mean age was 63.93 (SD 9.49) years. Educational background of primary school or less accounted for 58.5% of patients; patients who were married or had a partner accounted for 89.5%; and urban residents accounted for 45.8%.

As shown in Table 2, 1694 (25.74%) patients have four or more risk factors. Hypertension was the most common risk factor (78.51%), followed by overweight or obese smoking (61.58%) and dyslipidemia (54.38%) (Table 2).

**Figure 1:**
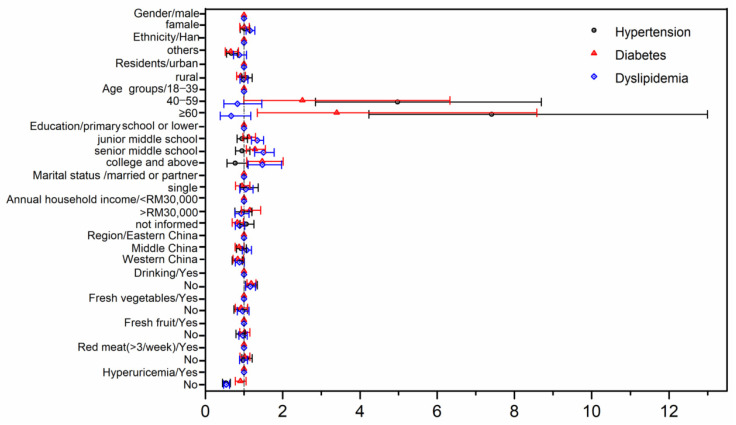
Adjusted (adjusted other different factors including gender, ethnicity, residents, age, marital status, and annual household income) odds ratios and 95% confidence intervals for hypertension diabetes dyslipidemia in different target groups.

Figure 1 shows the adjusted odds ratios and 95% confidence intervals for hypertension, diabetes, and dyslipidemia in different target groups.

Drinking stroke patients had a lower rate of hypertension, diabetes, and dyslipidemia. Patients with hyperuricemia had a higher rate of hypertension and dyslipidemia than no-hyperuricemia patients. No difference was found between red meat consumption more than three times per week, or eating fresh vegetables or fresh fruit every day on hypertension, diabetes, and dyslipidemia.

As shown in Table 3, 73.62% of patients knew they had hypertension before the survey, 70.19% had taken treatment measures, and only 919 stroke patients had their blood pressure controlled within the normal range. The awareness rate of diabetes was 66%, 70.19% had taken treatment measures for diabetes, and 34.51% of patients had their blood glucose controlled within the normal range. The awareness rate of dyslipidemia was 42.37%, 29.4% had taken treatment measures for diabetes, and 20.07% of patients had their blood glucose controlled within the normal range.

As shown in Table 4, the majority of hypertension-treated patients took medicine as medically advised (91.31%), 58.88% of them controlled their diet, 45.78% increased exercise, and 73.99% monitored blood pressure. A total of 78.24% and 34.71% of diabetes-treated patients were on oral antidiabetic medications or injected insulin, 85.77% were on diet control, and 78.24% were monitoring blood sugar. Among the treated dyslipidemic patients, 80.61% took medicine as medically advised, 77.57% controlled their diet, 56.46% increased their exercise, and 40.3% regularly monitored blood lipids.

## 4. Discussion

This study describes the prevalence and control of stroke risk factors in Chinese stroke patients. Our data show that about a quarter had four or more stroke risk factors. The prevalence rates of hypertension, overweight or obese and dyslipidemia were ranked as the top three stroke risk factors, with 78.51%, 61.58% and 54.38%, respectively. In our study, stroke patients had a high rate of conventional stroke risk factors, which is consistent with other studies [23,24].

Hypertension is the most common risk factor in patients with prevalent stroke, and more than two-thirds of stroke patients have hypertension which consists of other countries [25,26,27]. Hypertension is the most important metabolic risk factor of stroke recurrence [28]. The risk of stroke recurrence in patients with hypertension is significantly higher than in non-hypertensive patients [29,30,31]. The hypertension awareness, treatment and control rates in stroke patients were 73.62%, 70.19%, 17.79%. These rates were higher than the hypertension awareness, treatment and control rates of 43.8%, 39.2% and 13.8%, respectively, found in the 2015 China Health and Nutrition Survey [32] and the control rate was higher than hemorrhagic stroke patients in a survey, which 80.9% knew their hypertensive condition, and only 12.0% had blood pressure lower than 140/90 mmHg. In this study, the awareness rate of hypertension in patients with hypertensive stroke was similar to the treatment rate, indicating that most patients with hypertension awareness would take treatment measures, especially taking antihypertensive drugs (91.31%). However, the low control rate indicates that the current simple drug treatment cannot control the blood pressure of stroke patients within a reasonable range, and stroke patients should take healthy diet, increase physical activity and other comprehensive measures to control blood pressure.

The prevalence of diabetes among stroke patients was 21.75%, much higher than the findings reported in the national surveys conducted in China (11.6%) [33], and similar with a research of acute ischemic stroke patients in 40 hospitals in China (24.1%) [7]. 69.74% of patients were aware of their condition of diabetes, and less than two-thirds patients have treated diabetes, and the control rate was about one-third. Controlling diabetes can prevent both primary and secondary stroke and may reduce mortality too [23]. Diabetes promotes atherosclerosis and increases the risk of stroke, and the incidence of stroke in diabetic patients is 1.5 to 3 times that in non-diabetic individuals [34,35,36,37,38]. Most treated diabetes patients take oral medications or insulin injections, control diet, monitoring blood glucose, and the percentage of increased exercise was relatively low. Dyslipidemia is also a major risk factor for cardiovascular disease and stroke. The proportion of stroke patients with dyslipidemia was 54.38% was higher than the prevalence of China adults 41.9%. The awareness, treatment and control rates of dyslipidemia were 42.37%, 29.4% and 20.07%. higher than the rate of China adults 24.4%, 8.8% and 4.3%.

The major mechanism is dyslipidemia leading to stroke is atherosclerosis [39,40]. Several trials have reported that lipid-lowering drugs such as statins can reduce the risk of ischemic stroke [39]. The present study indicates that suboptimal dyslipidemia awareness, treatment and control are still major problems for stroke patients in China. In this study, 29.4% patients with dyslipidemia were treated, and 80.61% of them took lipid-lowering drugs as prescribed by the doctor. In a Hong Kong study of 466 patients with ischemic stroke, a median follow-up period of 8.7 years, had a recurrent incidence of statin medication of 23.8%. The rate of statin medication in patients with non-recurrent cerebrovascular event is 43.5% [41]. It shows that the treatment rate of stroke patients in China needs to be improved. The present study indicates that suboptimal dyslipidemia awareness, treatment and control are still major problems for stroke patients in China.

Awareness plays a role in the treatment and control of stroke risk factors. In this study, the awareness rate of diabetes and dyslipidemia in stroke patients is relatively low. Therefore, it is necessary to strengthen the health services, publicity and education of hypertension prevention and treatment for stroke patients in China. Previous studies have shown that health-education can improve the awareness rate of risk factors in patients [42,43].

Physical activity can reduce the risk of recurrent stroke occurrence and improve health. Appropriate physical activity can reduce the decline in the body’s motor skills and play a key role in preventing new complications [44]. The incidence of overweight or obesity in this survey was 61.58%, indicating that most stroke patients should control their weight. Risk factors such as atrial fibrillation and family history of stroke are also important factors affecting stroke recurrence. In this study, the proportion of stroke patients with atrial fibrillation and family history is 4.47% and 17.43%, respectively. These patients should improve their awareness of stroke prevention and treatment to prevent stroke recurrence. In the multivariate analysis, we found drinking was associated with lower hypertension, diabetes, and dyslipidemia. Blood pressure and diabetes are lower among individuals with moderate drinking than nondrinkers, but heavy drinking is associated with an increase in blood pressure [45,46,47]. However, the association between drinking and hypertension, diabetes, and dyslipidemia in stroke patients still needs further research.

Hyperuricemia was associated with higher hypertension, diabetes, and dyslipidemia; this result is consistent with the results of other studies [48,49,50]. Hyperuricemia stimulates the renin-angiotensin system and blocks the release of nitric oxide (NO) from endothelial cells, which leads to renal vasoconstriction and hypertension [49]. Red meat is a major food source of protein and fat, but there is a positive association between processed red meat intake and hypertension [51] and type 2 diabetes mellitus [52]. Although no effect of frequency of consumption of fruits and vegetables on stroke risk factors was found in this study, fruits and vegetables are rich in vitamins, minerals, and other nutrients, which play an important role in the rehabilitation of stroke patients and the reduction in stroke risk factors [53].

Studies show that compared with developed countries, the quality and quantity of stroke prevention and rehabilitation in China are insufficient [54]. With the increase in the number of stroke patients, how to control stroke risk factors in the community is very important. This cross-sectional study involved a large representative sample of the Chinese population. As far as we know, this study was the most recent investigation of stroke-risk factors of stroke patients in China. In this study, we described and analyzed the risk factors of community stroke patients in China, providing evidence for further management and control of stroke risk factors in patients with stroke.

The present study has several limitations. First, this study was a cross-sectional study, the use of self-reported stroke history, atrial fibrillation status, socioeconomic and lifestyle variables, and disease and medication adherence measurements in our results could be overestimated or underestimated. Second, stroke history was also based on self-report and was not confirmed by review of medical records. Third, since the number of hemorrhagic stroke patients in this study was small and the risk factors for stroke recurrence were roughly the same, there was no discussion by type. In future studies, we plan to follow-up with prospective participants, collect information about mortality or cause of death, and investigate stroke incidence in participants.

## 5. Conclusions

The most common risk factors for community stroke patients in China are hypertension, dyslipidemia, and overweight or obesity. The stroke community patients’ suboptimal awareness and treatment of hypertension, and suboptimal awareness, treatment, and control of diabetes, and dyslipidemia are significant problems in China.

## Figures and Tables

**Table 1:** Characteristics of stroke patients (*n* = 6580) of the China National Chronic Disease and Nutrition Survey (2015) in China (%).

Characteristics		Proportion	χ^2^	*p*
Gender			31.879	<0.001
	Male	53.48		
	Female	46.52		
Residence			47.320	0.071
	Urban	45.76		
	Rural	54.24		
Ethnicity			16,160.849	<0.001
	Han	92.80		
	Zhuang	0.46		
	Manchu	1.64		
	Others	5.11		
Age group			4852.297	<0.001
	18–39	0.1		
	40–59	0.7		
	≥60	4.8		
Education			4690.508	<0.001
	Primary school or lower	58.48		
	Junior middle school	26.91		
	Senior middle school	11.25		
	College and above	3.36		
Marital status			14,719.590	<0.001
	Never married	0.70		
	Married/partner	89.51		
	Divorced or separated	0.93		
	Widowed	8.86		
Region			220.155	<0.001
	Eastern China	37.6		
	Middle China	37.7		
	Western China	24.7		
Current smoker			1773.413	<0.001
	Yes	24		
	No	76		
Alcohol use			1162.729	<0.001
	Yes	29		
	No	71		
BMI Group			3451.943	<0.001
	Underweight	2.60		
	Normal weight	35.82		
	Overweight or obese	61.58		

Abbreviations: BMI, body mass index.

**Table 2:** Prevalence of risk factors of stroke patients in China (%).

Risk Factors	Total	Men	Women	χ^2^	*p*
Hypertension	78.51	78.20	78.86	0.422	0.516
Dyslipidemia	54.38	52.77	56.22	7.867	0.005
Diabetes	21.75	20.49	23.2	0.062	0.008
Lack of exercise	16.35	16.43	16.27	0.029	0.865
Atrial fibrillation	4.47	4.26	4.7	0.749	0.387
Overweight or obese	61.58	58.99	64.55	21.390	<0.001
Smoking	24.04	40.3	5.36	1094.201	<0.001
Family history of stroke	17.43	16.79	18.16	2.133	0.144
Four or more risk factors	25.74	34.27	15.94	287.674	<0.001

**Table 3:** Awareness, treatment, and control rate of hypertension, diabetes, and dyslipidemias in stroke patients in China (%).

	Awareness Rate			χ^2^	*p*	Treatment Rate			χ^2^	*p*	Control Rate			χ^2^	*p*
	Total	Men	Women			Total	Men	Women			Total	Men	Women		
Hypertension (*n* = 5166)	73.62	72.2	75.23	6.062	0.014	70.19	68.35	72.29	9.523	0.002	17.79	17.77	17.81	0.002	0.967
Diabetes (*n* = 1431)	69.74	66.99	72.54	5.212	0.022	65.83	62.55	69.15	6.933	0.009	34.59	32.32	36.9	3.324	0.068
Dyslipidemia (*n* = 3578)	42.37	38.13	46.95	28.479	<0.001	29.4	26.01	33.06	21.403	<0.001	20.07	17.66	22.66	13.912	0.000

**Table 4:** Medication adherence and other methods to control risk factors of stroke in Chinese stroke patients with hypertension, diabetes, or dyslipidemia (%).

		Sex		χ^2^	*p*
	Total	Men	Women		
Hypertension (*n* = 3626)					
Take medicine as medically advised	91.31	91.17	91.46	0.094	0.760
Diet control	58.88	56.46	61.49	9.462	0.002
Increase exercise	45.78	46.68	44.81	1.267	0.260
Blood pressure monitoring	73.99	74.00	73.93	0.000	0.989
Diabetes (*n* = 942)					
Oral medications	78.24	77.16	79.23	0.588	0.443
Insulin injection	34.71	38.36	31.36	5.075	0.024
Diet control	85.77	83.15	88.19	4.892	0.027
Increase exercise	57.86	60.53	55.4	2.542	0.111
Blood glucose monitoring	78.24	80.49	76.17	2.573	0.109
Dyslipidemia (*n* = 1052)					
Take medicine as medically advised	80.61	79.92	81.20	0.273	0.601
Diet control	77.57	76.19	78.73	0.972	0.324
Increase exercise	56.46	54.87	57.82	0.928	0.335
Blood lipid monitoring	40.3	36.65	43.41	4.967	0.026

## Data Availability

The data are not allowed to be disclosed according to the National Institute for Nutrition and Health, Chinese Center for Disease Control and Prevention.
